# Identifying Overlapping and Hierarchical Thematic Structures in Networks of Scholarly Papers: A Comparison of Three Approaches

**DOI:** 10.1371/journal.pone.0033255

**Published:** 2012-03-27

**Authors:** Frank Havemann, Jochen Gläser, Michael Heinz, Alexander Struck

**Affiliations:** 1 Institut für Bibliotheks- und Informationswissenschaft, Humboldt-Universität zu Berlin, Berlin, Germany; 2 Zentrum Technik und Gesellschaft, Technische Universität Berlin, Berlin, Germany; University of Namur, Belgium

## Abstract

The aim of this paper is to introduce and assess three algorithms for the identification of overlapping thematic structures in networks of papers. We implemented three recently proposed approaches to the identification of overlapping and hierarchical substructures in graphs and applied the corresponding algorithms to a network of 492 information-science papers coupled via their cited sources. The thematic substructures obtained and overlaps produced by the three hierarchical cluster algorithms were compared to a content-based categorisation, which we based on the interpretation of titles, abstracts, and keywords. We defined sets of papers dealing with three topics located on different levels of aggregation: *h-index*, *webometrics*, and *bibliometrics*. We identified these topics with branches in the dendrograms produced by the three cluster algorithms and compared the overlapping topics they detected with one another and with the three predefined paper sets. We discuss the advantages and drawbacks of applying the three approaches to paper networks in research fields.

## Introduction

Over the last years, increasing attention has been paid to the detection of overlapping substructures in networks. This focus is motivated by the observation that many real-world structures cannot be correctly represented by disjoint node subsets of networks. Scientific fields or, more generally, thematic structures in science are a case in point. The delineation of scientific fields is a pertinent problem of science studies in general and bibliometrics in particular (cf. e.g. van Raan, 2004, p. 39 [1]). Bibliometric research has shown that clusters in networks of papers do not have natural boundaries (cf. Zitt *et al.*, 2005 [2]). This is why fields must be delineated by applying thresholds for parameters. These thresholds cannot be derived from theoretical considerations. They must be chosen arbitrarily and are commonly justified in terms of ‘good structures’ for the purÂ­poses of the analysis at hand (cf. e.g. references [3], [4]).

However, the problem of delineation might be a consequence of the overlap of thematic structures. The overlap of themes in publications is well known to science studies. Sullivan *et al.* (1977, p. 235) [5] observed that in the literature of the field of weak interaction half of the references were articles outside the specialty. Amsterdamska and Leydesdorff (1989, p. 461) [6] provide an example of an article that targeted two different specialties at once. If disjoint clusters of co-cited sources (Marshakova 1973 [7], Small 1973 [8]) are projected forward to their citing papers, the clusters of citing papers inevitably overlap–a phenomenon that has never been explored by bibliometrics. Taken together, these observations suggest that the sciences consist of numerous fields of different sizes that partially or totally overlap, i.e. feature hierarchies as well as mutually overlapping ‘neighbours’ with fuzzy boundaries.

If thematic structures have boundaries that are hidden by their overlaps, delineation is not impossible in principle but rather depends on tools that enable the identification of overlapping fields and topics.

So far, only one such tool, namely co-citation analysis (hard clustering of papers according to the relative frequency of their joint citation by other papers), has been applied to the delineation task. However, this method assumes disjoint source clusters and locates thematic overlaps only in citing papers. This unrealistic assumption makes it unsuitable to detect overlapping topics.

The aim of this paper is to introduce and assess three algorithms for the identification of overlapping thematic structures in networks of papers. We derived these algorithms from three recently proposed approaches to the detection of overlapping and hierarchical substructures in networks–which in network analysis are called *communities*. For a concise description of the current state of finding communities in networks see the introduction of reference [9], for a recent review of methods which deliver overlapping communities see reference [10]. Our selection and specification of the general approaches is based on the assumption that the thematic substructures both overlap and build hierarchies.

We further had to take into account the information utilised by the different approaches. Thematic structures can be determined top-down using global information or bottom-up using either global and local or only local information. In our case, these different approaches correspond to different ways in which scientific perspectives are used in the construction of thematic structures. Since the production of contributions to scientific knowledge is based on the interpretation of that knowledge by individual producers [11], thematic structures in paper sets are always constructed from the individual perspectives of the authors. A bottom-up approach using only local information enables the reconstruction of thematic structures from the perspective of those contributing knowledge to these themes. The use of global information in the top-down or bottom-up construction of thematic structures, e.g. by spectral and modularity-based methods (cf. Fortunato's 2010 review paper, p. 41, p. 27 [12] and also reference [13]), is akin to including the perspective of ‘outsiders’, i.e. of authors/papers not contributing to the specific topic. Such a ‘democratic’ procedure can be justified as well but is likely to lead to different results (for an attempt to justify the global perspective see Klavans and Boyack, 2011 [14]).

These considerations made us select three approaches that enable the identification of overlapping and hierarchical structures in networks on the basis of local information. A first approach starts from hard clusters obtained by any clustering method and fractionally assigns the nodes at the borders between clusters to these clusters (cf. e.g. Wang *et al.*, 2009 [14]). Another approach is based on a hard clustering of links between nodes into disjoint modules, which makes nodes members of all modules (or communities) that their links belong to (cf. e.g. Ahn *et al.*, 2010 [15]). The third approach constructs *natural communities* of all nodes, which can overlap with each other, by applying a greedy algorithm that maximises local fitness (cf. e.g. LanciÂ­chinetti *et al.*, 2009 [16]).

We introduce the three approaches to finding overlapping communities and explain their basic mechanisms with a simple example, namely the social network of 34 members of a karate club analysed by Zachary (1977) [17] (using the unweighted graph). Members of the karate club were asked about friendship ties. The network turned out to have two central actors who, after the split of the original club, founded separate new clubs. Authors who implemented algorithms based on the three approaches applied them to the network described by Zachary.

The comparative analysis applies the algorithms to a network of 492 bibliographically coupled papers published 2008 in six information-science journals. The use of information-science papers enabled the construction of paper sets of selected topics by manually assigning papers to the topics *h-index*, *webometrics*, and *bibliometrics* on the basis of titles, abstracts, and keywords. The clustering solutions and the overlap of modules were then assessed by comparing them to the paper sets. On the basis of this comparison we discuss advantages and disadvantages of the three algorithms.

## Methods

### Reconceptualising Communities in Networks as Fuzzy Sets

In network analysis, communities are understood as cohesive subgroups of nodes separated from the rest of the graph i.e. as groups of densely interconnected nodes that are less densely connected to other nodes. Most community definitions are based on these two aspects, i.e. cohesion and separation [12] (pp. 83–87). Therefore, algorithms for the detection of communities in the above-described sense are based on definitions of cohesion and separation, too [18]. Owing to the continuous nature of the two properties, communities cannot be detected unequivocally. Instead, structures of varying ‘communityness’ can be identified [19].

The definition of communities in networks by cohesion and separation is used in our paper thrice, namely (a) for the identification of interesting communities in the dendrograms; (b) in the fitness function used by two algorithms; and (c) in the characterisation of the fuzzy communities constructed by the algorithms.

Cohesion and separation can be measured in different ways. For hierarchies of communities, both cohesion and separation can be measured directly in the dendrogram. The simplest measure of separation of a community is the dissimilarity level 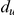 at which its branch in the dendrogram unites with another branch. The simplest measure of cohesion of a community is the dissimilarity level 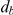 at which its branch in the dendrogram is build from two branches. A low value of 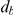 represents high cohesion.

Thus, high ‘communityness’ is characterised by a high level of 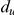 and a low level of 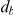. This is why the long branches in dendrograms are commonly considered to be important ones. They have large differences 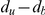, i.e. are stable over relatively large dissimilarity intervals. Using this difference, we can order branches with respect to their quality as communities, i.e. combined cohesion and separation.

In our experiments, stability is negatively correlated with community size. Many small branches are very stable and many larger branches are very unstable. In order to find ‘interesting’ communities, we plot branch length 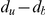 over community size and identify communities that are unusually stable for their size, i.e. are represented by branches far from the axes of the plot.

Another approach to community delineation associates cohesion with high internal and separation with low external degrees of community members. The internal degree 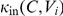 of a node 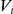 is defined as the sum of weights of edges linking this node with nodes in community 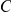, its external degree 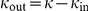, where 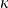 is the node's total degree. Radicchi *et al.* (2004) [20] define a *community in the strong sense* as a set of nodes all of which have higher internal than external degrees. For a *community in the weak sense* they only demand that the sum of internal degrees exceeds the sum of external degrees. These sums are usually referred to as the internal and external degrees of community 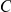:
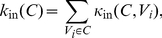
(1)

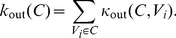
(2)


A disadvantage of this measurement is that there can be coherent subsets of nodes which are separated from the rest of the graph but do not match the weak definition. For example, Ahn *et al.* (2010, p. 1) [15] state that the weak definition of communities “break[s] down when overlap is pervasive”, i.e. when “overlap can exist for each and every node”, because “[w]hen overlap is pervasive, counterintuitively, each community has many more external than internal connections.” (However, in our tests of the three algorithms on a network of papers we obtained communities that match the weak definition, see below, section *Results*.)

The internal and external degrees of a community can be used to define its fitness (see the approaches ‘natural communities’ and ‘fuzzification’ for applications). By combining cohesion and separation, the fitness measure evaluates the quality of a community in a similar way as 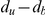 does based on a community's branch in a dendrogram.

When applied to overlapping communities, the measures used in the delineation of weak communities must take the nature of overlaps into account. Following Steve Gregory (2011) [21], we distinguish between crisp and fuzzy overlapping communities. If a network has crisp overlapping communities, nodes either belong or don't belong to a community. Overlapping communities are fuzzy if individuals' grades of membership vary. This type of structure is appropriate for the relationship between papers and topics because most papers cover several topics in varying intensities, which led us to the application of fuzzy-set theory. For basic definitions in fuzzy-set theory used here we refer to the Supporting Information S1.

Fuzzy set theory operates with membership grades that are real numbers between zero and one but does not assume that a node's grades of membership in different sets sum up to unity. A node could also be a full member in more than one community.

To determine whether a fuzzy community 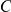 is a community in the weak sense we have to redefine its internal and external degree 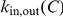 by weighting the degrees with node membership grades. With 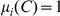 if 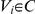 and 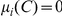 otherwise, we can rewrite the definitions given above for crisp communities as
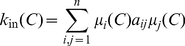
(3)and
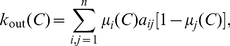
(4)where 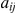 is the weight of edge 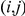 and 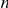 the graph size. These formulae can also be used for a fuzzy community 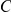 if 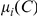 is identified with node's 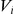 membership grade in 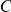. Then 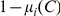 is its membership grade in 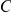
's fuzzy complement. Fuzzy set 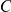 is a community in the weak sense if 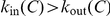.

### Constructing Natural Communities of Nodes

#### The Approach

**Figure 1:**
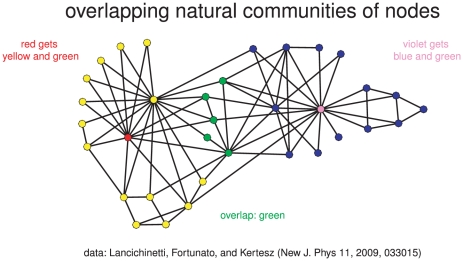
Natural communities. Karate club graph with overlapping communities of two nodes (red and violet).

A *natural community* of a node is a community that is constructed by a ‘greedy’ algorithm which evaluates the inclusion of neighbouring nodes into the community using an appropriate metric or fitness function. If a community with a neighbour node is fitter than without it, the neighbour will be included, which leads to a stepwise growth of the natural community. The essence of this local approach is that independently constructed natural communities of nodes can overlap. Figure 1 shows two overlapping communities of karate club members. On the left-hand side, the red node's community has all yellow and green nodes as its members. On the right-hand side, the violet node's community has all blue and green nodes as members. Thus, we have five (green) nodes in the overlap of both natural communities.

The idea to construct overlapping communities as sub-graphs which are locally optimal with respect to some given metric was first published by Baumes *et al.* (2005) [22]. It can be implemented in several ways one of which was tested by Baumes *et al.* in the same year [23].

Lancichinetti *et al.* (2009) [16] combined the concept of locally optimal sub-graphs with the idea of variable resolution to enable their algorithm to reveal hierarchical community structures. They introduced a resolution parameter into their fitness function. Higher resolution results in smaller, lower in larger natural communities. The fitness function includes only local information. It is defined as the ratio of the sum of internal degrees 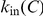 to the sum of all degrees 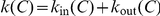 of nodes in a community 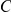. The denominator is taken to the power of 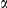, the resolution parameter:
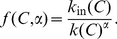
(5)


Figure 1 displays a cover of the karate-club network obtained by Lancichinetti *et al.* with a stochastic version of their algorithm for the resolution interval 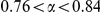. Their LFM (**l**ocal **f**itness **m**aximisation) algorithm has to be repeated for all resolution levels of interest.

The construction of a scientific paper's natural community in a similarity network of papers can be interpreted as the construction of its thematic environment from its own ‘scientific perspective’. This idea is attractive from a conceptual point of view because it mimics the way in which scientists apply their individual perspectives when constructing their fields. This is why locality is a realistic assumption for topic extraction in paper networks.

At the same time, the strictly local approach enables the local exploration of networks which are too big for global analysis like the Web or the complete citation network of scientific papers. A node's natural community is a local structure that can be constructed without knowing the whole graph. The idea to find local community structures without knowing the whole graph by using a greedy local cluster algorithm goes back to Clauset (2005) [24]. His procedure can also be used to construct overlapping graph modules [25]. In contrast to the resolution-depending fitness function of Lancichinetti *et al.* (2009) [16] Clauset evaluated modules with a function that does not depend on resolution.

#### MONC Algorithm

MONC [26] uses ideas from Lancichinetti *et al.* (2009) [16] but replaces their numerical approach by a faster and more precise parameter-free analytical solution. The specific form of the fitness function itself is the only arbitrary presetting of the algorithm. Other resolution-dependent fitness functions are possible [27] (p. 8).

Lancichinetti *et al.* proposed an algorithm which rests on a greedy expansion of natural communities of nodes by local fitness maximisation (LFM algorithm). Communities of different nodes can overlap each other. The size of a natural community of a node depends on resolution 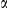. LFM has to be repeated for each relevant resolution level to reveal the hierarchical structure of the network. Our parameter-free MONC algorithm exactly calculates resolution levels at which communities change by including a node that improves their fitness. To save further computing time, MONC **m**erges **o**verlapping **n**atural **c**ommunities when they become identical during the iteration process [26].

Similar to Lee *et al.* (2010) [28]–who tested a variant of LFM–we found that using cliques as seeds gives better results than starting from single nodes. While Lee *et al.* use maximal cliques (i.e. cliques which are not sub-graphs of other cliques), we optimise clique size by excluding nodes that are only weakly integrated [26] (p. 6). MONC assigns each node to the seed clique whose fitness it improves at maximal resolution and then constructs a natural community as an ordered set of nodes entering the community at decreasing levels of resolution. For a more detailed description of MONC we refer the reader to the Supporting Information S1.

From MONC results, we construct *fuzzy natural communities* of nodes, i.e. fuzzy sets in which each node of the graph has a membership grade. Each fuzzy natural community represents its seed node's perspective on the whole network. Since the emphasis on local perspectives lets MONC construct many natural communities that are very similar, the fuzzy natural communities are hard-clustered hierarchically using the fuzzy-set Jaccard index as a similarity measure for, e.g., single-linkage clustering. Branches in dendrograms derived from MONC results do not represent disjoint sets of nodes but overlapping fuzzy communities.

#### MONC Post-Processing

Greedy algorithms which locally maximise a resolution depending fitness (or density) function can reveal hierarchies of overlapping modules. Lancichinetti *et al.* (2009, pp. 7–9) [16] have successfully tested their LFM algorithm on a simple benchmark graph with two hierarchical levels.

MONC (like LFM) needs some postprocessing to reveal a graph's hierarchy. We successfully tested the following procedure for detecting a graph's hierarchy from MONC results. A node's membership grade in a community depends on the resolution level at which it becomes a member (cf. next subsection). With this definition communities become fuzzy sets over the universe of all nodes. Two communities are similar if their fuzzy intersection is large. As a relative measure, we use the fuzzy Jaccard index to define the similarity of two natural communities. Then communities can be clustered by any hard-cluster algorithm to reveal the graph's hierarchy.

Here we should add a comment. We construct a node's perspective on the whole graph i.e. its natural community as a fuzzy set over the universe of all nodes. We hierarchically cluster the fuzzy sets which is equivalent to node clustering based on a variant of the concept of structural equivalence [12] (p. 86). Nodes are structurally equivalent if their neighbourhoods are equal, they are structurally similar if their neighbourhoods are similar in some sense. We operationalise structural similarity of two nodes as the fuzzy Jaccard index of their fuzzy natural communities representing their perspectives on the whole graph. Despite the equivalence of our method to the concept of structural similarity of nodes we insist on the definitions given above: we do not cluster nodes but their fuzzy natural communities.

In our earlier paper [26] (section 4.3, p. 16) we discussed a post-processing different from the one applied here. At that time we tested MONC on non-hierarchical benchmark graphs and had to chose a resolution level. After determining all communities existing at this level we found many near-duplicates which will merge at some lower level of resolution. From each set of near-duplicate communities we constructed a *consensus module* [26] (pp. 21–22).

#### MONC Grades of Membership

MONC's greedy expansion of seeds can be discussed in terms of ‘hosts inviting guests’ to their communities. Each node 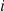 of the (connected) graph is ‘invited’ to each community 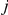 at some level of inverse resolution 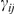. To construct fuzzy communities with various grades of node membership we propose to define the membership grade of node 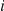 in the community of node 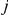 as
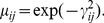
(6)Using the decreasing exponential function of squared 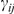 (as in the density function of the normal distribution) ensures that

1. the host is full member in its own community because it is a member of its own natural community at infinite resolution (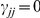, i.e. 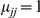),
2. ‘late guests’ get lower grades, and
3. the ‘first guests’ get membership grades near one (the function starts from one, its derivation from zero).

We assume that the dendrogram of fuzzy natural communities reflects the graph's hierarchical structure. For each branch we define a community as the fuzzy union of all fuzzy sets of the branch's nodes. This means that all host nodes of the branch are full members of the branch community. This definition ensures the hierarchical order of branches: if two branches unite then their communities are fuzzy subsets of their fuzzy union. Thus, each branch of the dendrogram of fuzzy natural communities, i.e. each vertical line, represents a fuzzy community.

### Fuzzy Node Communities from Hard Link Clustering

#### The Approach

**Figure 2:**
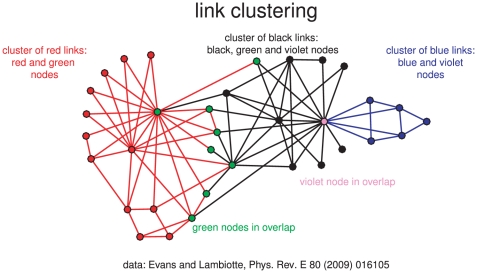
Hard clusters of links. Karate club graph with overlapping node communities induced by three hard link clusters.

If links instead of nodes are clustered, nodes with more than one link can be fractional members of clusters, as Figure 2 shows for the karate club. For example, vertex 1 (violet point) has four edges belonging to one and twelve edges belonging to another hard cluster of links. Thus, it has membership grades 4/16 and 12/16, respectively, in the two clusters.

For clustering links we need a measure of link similarity. Restricting the analysis to connected links, Ahn *et al.* (2010, eq. 2, p. 5 [15]) chose the Jaccard index of neighbourhoods of the two nodes at the not connected ends of the two links (a node itself is included into its neighbourhood).

In a different approach to link clustering, Evans and Lambiotte (2009) [29] used the line graph of an undirected graph. To get a graph's line graph first a bipartite graph of the graph's nodes and edges is constructed by putting an edge node on each edge. The bipartite graph can then be projected onto the line graph, a graph where nodes and edges have interchanged their roles.

Recently Ball *et al.* (2011) [30] successfully tested an algorithm which finds overlapping node communities with a generative stochastic model of hard link clusters. Kim and Jeong (2011) [31] applied the fast *Infomap* [32] algorithm to link clustering.

The clustering of citation links instead of papers is of high interest to bibliometrics because a citation is probably the conceptually most homogenous bibliometric unit. Since many references are referred to only once in a paper, it can be assumed that these links between the citing and the cited publication can be assigned to one theme. Even though there are many cases in which a paper cites a source for several different reasons, a citation link can be assumed to have a higher thematic homogeneity than a publication. Based on this assumption of homogeneity, citation links can be hard-clustered, which leads to overlapping clusters of papers. The membership grade of a paper to a module corresponds to the part of outgoing citation links of this paper within this link cluster.

We applied the **h**ierachical **l**ink **c**lustering (HLC) method suggested by Ahn *et al.* (2010) [15] to cluster citation links in the approximately bipartite network of papers and their cited sources. Ghosh *et al.* (2011) [33] have generalised HLC to tripartite graphs.

We did not consider the line-graph approach because it is not local (due to its use of modularity).

#### HLC Algorithm on Bipartite Citation Graphs

We consider the approximately bipartite network of papers and cited sources. Citation links between the two types of nodes can be hard-clustered, which leads to induced overlapping communities of papers (and also to communities of sources which, however, are not analysed here). The membership grade of a paper to a thematic community equals the fraction of its citation links belonging to the corresponding link cluster.

Links can be seen as similar if the neighbourhoods of their nodes overlap to a high degree. Thus, the Jaccard index of these neighbourhoods can be used as a similarity measure (cf. Ahn *et al.*, 2010, eq. 2, p. 5 [15]). We discuss the definition of similarity between links in a bipartite graph in terms of papers and cited sources. The neighbourhood of a paper 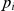 is the set of its references 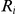, the neighbourhood of a cited source 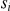 is the set of papers 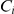 citing it. The neighbourhood 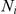 of citation link 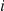 is then the union of these disjoint sets: 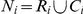.

Since papers might cite sources from the same year, some of the cited sources are also citing papers. However, in the 2008 volumes of six information-science journals we found less than one percent of citation links to about 60 papers (of 492) in these volumes. This means that only a small proportion of citation links were misclassified due to their incomplete neighbourhood.

The size of the intersect of two link neighbourhoods is given by
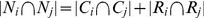
(7)and the size of their union by
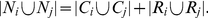
(8)The distance metrics used for clustering is then
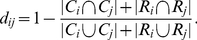
(9)


Ahn *et al.* calculate similarities only for link pairs which have a node in common because they “expect” disconnected link pairs to be less similar than pairs connected over a node [15] (p. 5). Since counterexamples disproving this assumption can be constructed, we decided to calculate similarities for all pairs of nodes. Such a procedure uses more information but is also more time-consuming.

Hard clustering of links can be done with any hierarchical clustering method. We tested four standard methods. The dendrograms of Ward and average clustering of citation links seem to reflect the graph's hierarchy more adequately than those of single-linkage and complete-linkage clustering. The latter two methods impose too low or too high restrictions, respectively, on finding clusters.

#### HLC Grades of Membership

As discussed above, the membership grades of nodes in HLC communities are already unambiguously determined by the algorithm itself. A node's grade is the portion of its links in the link cluster under consideration.

### Fuzzification of Hard Clusters

#### The Approach

The approach assumes that hard-cluster algorithms validly identify disjoint community cores which just need to be ‘softened’ at the borders. If this is the case, modifying a hard cluster by evaluating the inclusion of its nodes and neighbouring nodes with regard to some metric or fitness is a plausible method for constructing overlapping communities. The fitness balance of a node with respect to a cluster can then be used to decide about its membership and to calculate its membership grade. Thus, we construct fuzzy overlapping communities.

**Figure 3:**
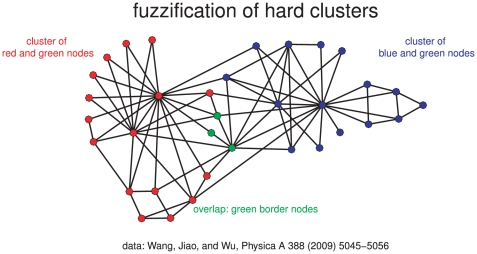
Fuzzification of hard clusters. Karate club graph with overlapping communities from two hard clusters.

Figure 3 shows the karate-club result Wang *et al.* (2009) [14] obtained with their implementation of the fuzzification approach, which they applied to two hard clusters.

#### Fuzzification and Membership Grades

A straightforward approach to making hard clusters overlapping and fuzzy is to redefine the membership grades of all nodes with links crossing borders. If some of a node's links end within cluster 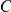 and some outside 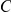 then its membership grade 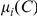 can be defined as
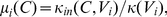
(10)where 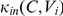 is the sum of weights of edges between vertex 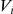 and vertices in 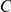 and 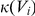 the sum of all its edge weights. We use this definition to calculate fractional grades after we constructed overlapping communities by fitness improvement. We also tested a definition of membership grades that uses the fitness balances of nodes at cluster borders. However, we used the definition by equation 10 because the result is better comparable to the results of the other two algorithms.

Since with equation 10 fractional memberships are calculated using non-fractional (zero or full) memberships of nodes as obtained by fitness evaluation, it would be interesting to see whether an iteration procedure converges.

For such an iteration we reformulate fractional assignment of membership grades. The definition in equation 10 is equivalent to calculating the new membership grade 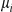 of node 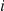 as the average of its neighbours' current grades. If the graph is weighted we have to weight a neighbour's grade with the link weight. Recalculating grades can therefore be done by multiplying the vector 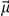 of zero-one grades 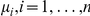 of a hard input cluster with a matrix 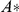, which is obtained from the adjacency matrix 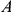 by normalising its rows to sum to one:
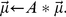
(11)The node's own grade could be included into the average by setting the diagonal of the adjacency matrix to one, 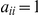. In both cases, the iteration according to equation 11 lets any initial grade vector 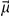 that is not orthogonal to the principal eigenvector of matrix 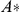 converge to that eigenvector. It can be shown that all components of the principal eigenvector of matrix 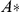 are equal. In fact, when we iterate the whole graph's grade vector, i.e. start from 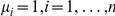, then averages of grades are always equal to one and the iteration does not change any grade. Thus, the iteration indeed converges but its result is trivial and cannot be used to assign meaningful membership grades to nodes.

Steve Gregory's COPRA algorithm for the construction of overlapping communities [34] also averages membership grades of neighbours in an iteration to obtain a node's grade. COPRA avoids the trivial solution of uniform memberships by deleting grades below a threshold [34] (p. 5). We did not yet test this method because we search for parameter-free algorithms.

#### Fuzzification Algorithm

We implemented an algorithm that evaluates border nodes of each hard cluster with regard to their connections with it. Border nodes have edges crossing the cluster's border and can be located inside or outside the cluster.

The algorithm uses an evaluation metric that is based on the fitness function defined by Lancichinetti *et al.* (2009) [14], [16] (see above, equation 5, page 4). For each border node of a cluster we calculate the cluster's fitness with and without this node. The fitness balance of a node with respect to a cluster determines its membership. Negative balance means exclusion from the cluster. We evaluate all border nodes of a cluster without changing it during the evaluation (in contrast to the greedy LFM algorithm which updates the community after deciding about a node's membership). The fitness-inherent resolution paraÂ­meter controls the extent of the overlap, where lower values cause a wider area to be considered for the inclusion into the former hard cluster. While MONC uses resolution levels to calculate membership grades, the fitness-inherent parameter is arbitrary here. Thus, we didn't apply it in our comparison and set 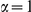.

In a second step the crisp overlapping communities are made fuzzy. The fractional membership grade of a node could be defined using its fitness balance as input but this did not lead to fuzzy communities that match the three predefined topics. Hence, we used a definition that ignores the value of (positive) fitness balances: The membership grade 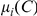 of vertex 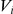 in community 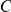 is defined by equation 10.

For a more detailed description of the fuzzification algorithm we refer the reader to the Supporting Information S1.

### Data

#### The Paper Network

We apply the three algorithms to a network of papers in the 2008 volume of six information-science journals with a high proportion of bibliometrics papers (for details of data see reference [26], papers downloaded from Web of Science).

We start from the affiliation matrix 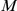 of the bipartite network of papers and their cited sources. Here we neglect that a few cited sources (less than one percent) are also citing papers in the 2008 volume. Link clustering is done with 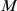 itself, the other two algorithms analyse a bibliographic-coupling network constructed from 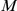 as follows. In the network of papers, two nodes (papers) are linked (bibliographically coupled) if they both have at least one cited source in common. To account for different lengths of reference lists we normalise the paper vectors of 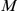 to an Euclidean length of one. With this normalisation, the element 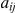 of matrix 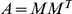 equals Salton's cosine similarity of bibliographic coupling between paper 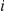 and 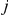.

The symmetric adjacency matrix 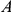 describes a weighted undirected network of bibliographically coupled papers. The main component of the network of 533 information-science papers 2008 (528 articles and five letters) contains 492 papers. Three small components and 34 isolated papers are of no interest for our cluster experiments.

#### Three Topics

For the evaluation of the three algorithms, we compare the topics they construct with three topics we identified ourselves. Using our knowledge of bibliometrics, we could identify three topics belonging to that field, namely 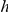-index, bibliometrics, and webometrics. The 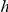-index is an indicator for the evaluation of a researcher's performance, which has been proposed by the physicist J. E. Hirsch in 2005. Since then, the use of the 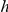-index for evaluating individual researchers, proposals for 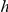-index derivatives and for 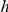-indices of journals or other aggregates of papers have been discussed in the literature. 46 of the 492 papers cite the 2005 paper by Hirsch, which is the most cited source in our sample. The 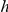-index is clearly an invention in the field of bibliometrics. About 200 other papers are also addressing bibliometric themes. For the purposes of this evaluation, we excluded analyses of patents from bibliometrics. In a smaller webometrics set, internet activities of (mainly academic) institutions and individuals are analysed.

**Figure 4:**
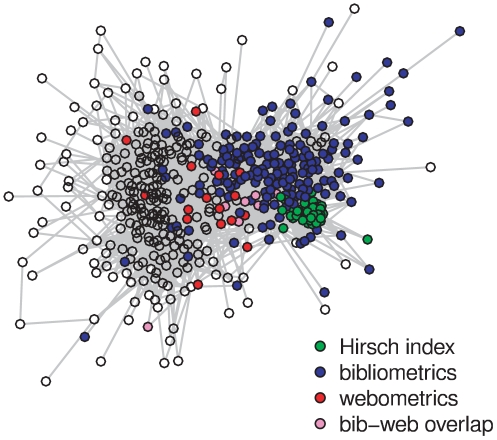
Information science 2008. Three topics and their overlaps in a network of 492 bibliographically coupled papers. Topics assigned manually to papers by inspection of their keywords, titles and abstracts. The nodes' colours correspond to four sets: (i) green to 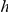-index, (ii) blue to bibliometrics without 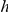-index and without webometrics, (iii) red to webometrics without bibliometrics, and (iv) violet to the overlap of webometrics and bibliometrics. Transparent nodes are papers dealing with other information-science topics, mainly with information retrieval and information behaviour.

We first assigned papers to the three topics on the basis of their keywords and subsequently checked the classification by inspecting titles and abstracts. This led to 42 papers assigned to the 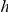-index and its derivatives, further 182 bibliometric papers not mentioning the 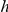-index in title or abstract, 24 webometric papers, and eight papers in the overlap between webometrics and bibliometrics. In Figure 4 we display the graph of the sample of 492 bibliographically coupled papers using the force-directed placement algorithm by Fruchterman and Reingold (as implemented in the **R**-package *igraph*, cf. http://www.r-project.org).

## Results

### MONC

**Figure 5:**
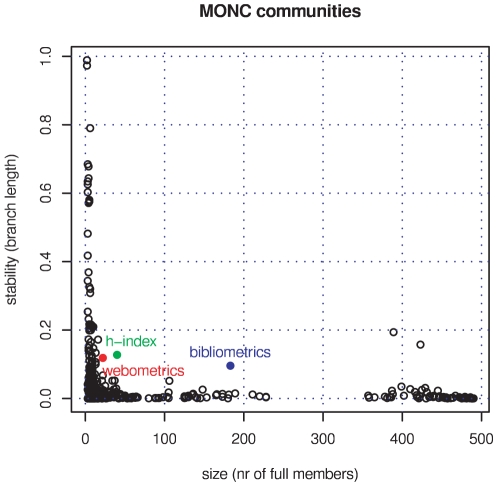
Stability over size of all MONC branch communities. Stable communities corresponding to our three topics in information-science papers 2008 are marked: bibliometrics (blue), webometrics (red), 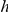-index (green).

For each branch community we plot its stability i.e. its branch's length 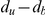 over community size, which is estimated by the number of full members (Figure 5, cf. also above, p. 2). Three of the outliers correspond to our predefined topics, and will be evaluated in comparison with results of the other algorithms (see below, section *Comparison of Identified Communities*). The two stable communities with about 400 full members unite bibliometrics, webometrics, information retrieval and some other smaller topics but do not include a set of less central graph nodes.

**Figure 6:**
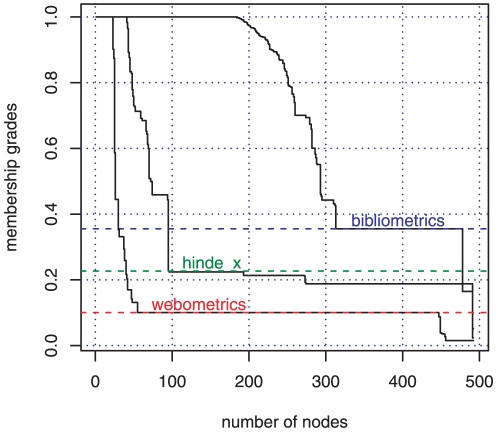
Plots of MONC communities of the three topics in information-science papers 2008: 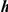-index, bibliometrics, webometrics. Coloured lines mark corresponding thresholds.

For better comparability, all membership grades below a threshold are set to zero. We derive the thresholds from plots of membership grades (Figure 6). These plots show that at some critical membership grade the node sets of each branch inflate to nearly the whole graph. We argue that this inflation marks the border of a community of a branch. We set the grade's threshold on a value that cuts the step curve at the last steepest gradient before inflation (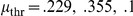 for 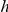-index, bibliometrics, and webometrics, respectively).

### Hierarchical Link Clustering

For all pairs of citation links from the 492 citing papers to all sources we determine link similarities. We restrict clustering to all 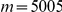 citation links to sources which are cited more then once.

Citation links to sources cited only once are excluded because all such links from the same reference list would be clustered at zero-distance level with one another and then merged with the link to the least cited source in their list. This cannot be justified on the basis of what is known about these links.

**Figure 7:**
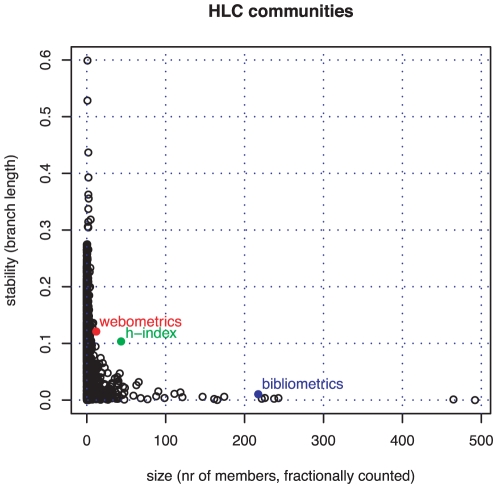
Stability over size of all 5004 HLC branch communities. Stable communities corresponding to our three topics in information-science papers 2008 are marked: bibliometrics as blue, webometrics as red, and 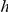-index as green point, respectively.

We applied the average-clustering method to this set. The corresponding dendrogram does not give a clear picture of the graph's hierarchy unless we re-parametrise the distance axis. We choose 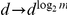 to de-skew distances 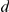. Using these rescaled data, we plot branch length over community size to find relatively stable and large communities (Figure 7). We measure community size by the sum of fractional membership grades of papers attached to the clustered citation links. Like in the MONC case, we find our three topics as exceptional points in the plot although the stability of the bibliometrics branch is only high in comparison with its predecessors and followers in the dendrogram.

### Fuzzification Algorithm

**Figure 8:**
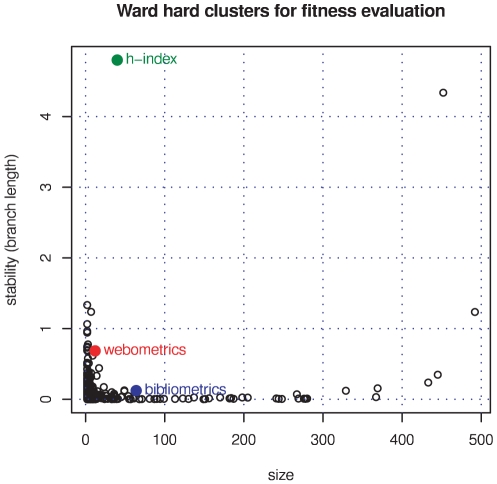
Stability over size of branch communities. Stable Ward clusters corresponding to our three topics in information-science papers 2008 are marked: bibliometrics as blue, webometrics as red, and 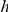-index as green point, respectively.

**Figure 9:**
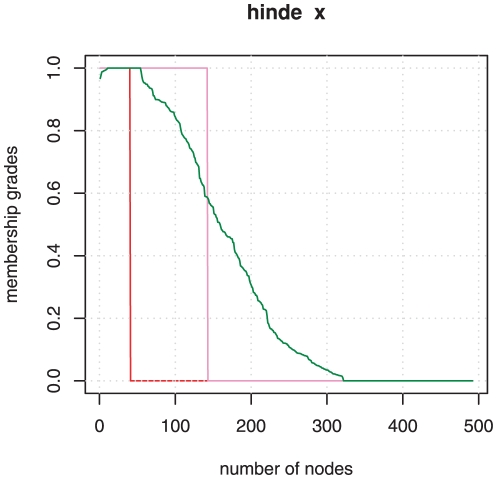
Membership distribution of 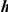-index topic in information-science papers 2008 determined by the fuzzification algorithm. The red step curve represents the initial hard cluster, the violet curve the members after fitness maximisation, and the green curve the grades of membership in the final fuzzy community.

We applied standard Ward and average clustering on the network of 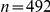 bibliographically coupled information-science papers (based on arc cosine of cosine similarity as the distance measure). Complete and single linkage failed to provide acceptable results as can be already deduced from the dendrograms. Average clustering also results in a dendrogram which is not easy to interpret. The Ward dendrogram shows a very stable and clear 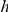-index cluster which is united with the rest of the graph in the last merging step (cf. Figure 8). Fitness-based optimisation with resolution 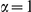 enlarges this cluster extremely and lowers precision without gain in recall with respect to the set of manually selected 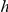-index papers. Figure 9 visualises how the original hard cluster is expanded and shows how membership grades of the final fuzzy community are distributed. Thus, fitness maximisation is not a successful strategy for this topic that has been well matched (by e.g. Ward) clustering already and is highly connected to its network environment.

If we omit fitness maximisation and only calculate fractional membership grades according to equation 10 the result is not better. Many external border nodes become partial members of the fuzzy 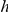-index community.

On the other hand, the hard bibliometrics cluster is much smaller than expected and needs fitness maximisation or at least fractional membership grades to match the topic.

### Comparison of Identified Communities

To compare the results we calculate fuzzy Salton's cosine of manually defined topics with fuzzy communities identified by the three algorithms considered. In addition, table 1 gives values of fuzzy precision and recall, the geometric mean of which equals the cosine. The fuzzy versions of cosine, precision, and recall are calculated with fuzzy set variants of intersection and set size.

**Table 1:** Topic matches by algorithms.

topic	MONC	HLC	fuzzy
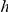 *-index*	.71	.93	.59
precision	.56	.91	.35
recall	.89	.95	1.00
*bibliometrics*	.79	.82	.83
precision	.72	.83	.87
recall	.86	.81	.80
*webometrics*	.58	.60	.46
precision	.53	.85	.45
recall	.64	.43	.47
*bib-web overlap*	.46	.29	.30
precision	.34	.24	.14
recall	.64	.36	.65

Fuzzy cosine indices, precision, and recall of paper sets and fuzzy communities (and of bibliometrics-webometrics overlap) found by the three algorithms.

**Figure 10:**
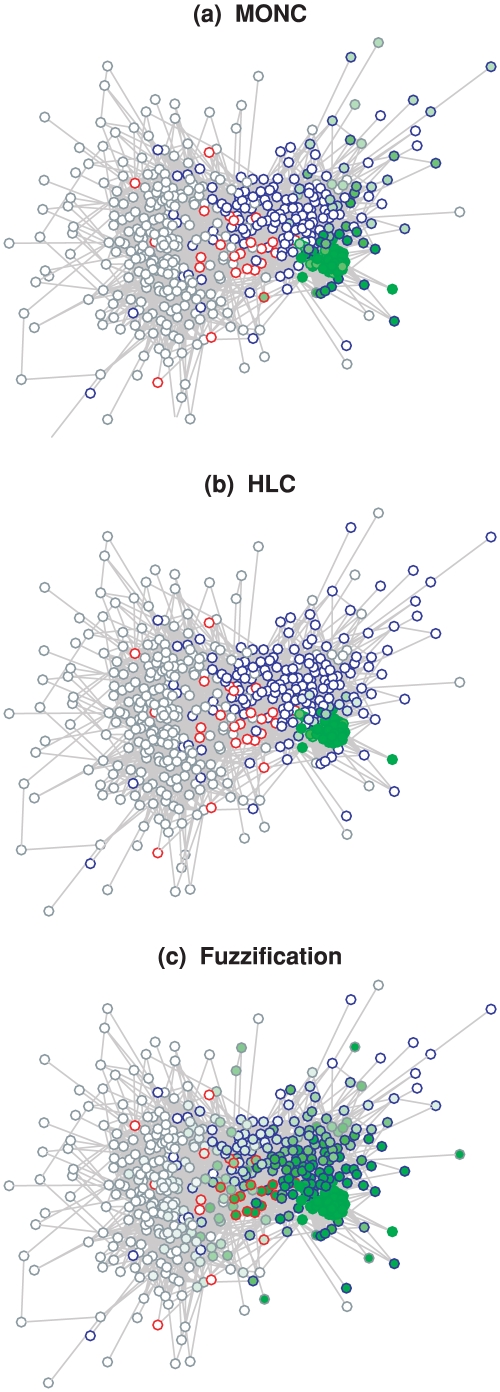
The 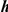-index communities constructed by the three algorithms in information-science papers 2008. Saturation of points correlates with membership grade. Colours of circles denote manually determined topics (green to 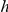-index, blue to bibliometrics without 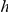-index and without webometrics, red to webometrics without bibliometrics, and violet to the overlap of webometrics and bibliometrics).

Figure 10 shows how the 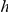-index topics identified by the three algorithms fit this topic as the pre-defined paper set.

Table 2 shows how fuzzy communities constructed by the algorithms overlap each other. In table 3 we list the fuzzy internal and external degrees, 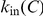 and 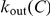 (cf. equations 3 and 4, p. 4), together with their ratio 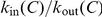 for each fuzzy topic community constructed by the three algorithms. Note, that for all three algorithms the values of 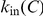 and 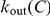 are calculated using cosine similarity of bibliographic coupling as link weights. All fuzzy communities are communities in the weak sense. The ratio can be interpreted as a measure of ‘communityness’.

**Table 2:** Community matching *between* algorithms.

	MONC	HLC	fuzzy
topic	HLC	fuzzy	MONC
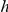 *-index*	.73	.60	.62
*bibliometrics*	.76	.84	.78
*webometrics*	.63	.46	.55
*bib-web overlap*	.51	.41	.43

Fuzzy cosine indices of fuzzy communities (and of bibliometrics-webometrics overlap) found by the three algorithms.

**Table 3:** Fuzzy 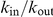 of communities.

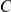	variable	MONC	HLC	fuzzy
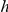 *-index*	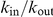	5.97	7.41	9.70
	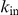	244.65	245.66	352.21
	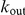	40.98	33.17	36.31
*biblio-*	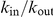	3.41	19.03	15.37
*metrics*	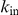	314.23	466.97	456.97
	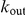	92.03	24.54	29.74
*webo-*	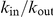	1.43	1.21	1.32
*metrics*	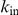	21.04	10.74	45.01
	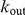	14.71	8.85	34.19

The ratio 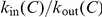, 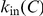, and 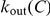 of fuzzy communities found by the three algorithms with regard to the bibliographic coupling graph of papers.

In order to test wether the three communities match the weak community definition also with regard to the bipartite graph of papers and cited sources we calculated 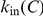 and 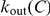 for all communities of papers and sources induced by the corresponding link clusters. Table 4 shows that in the full graph of papers and sources all three selected communities match the weak definition, even if 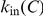 and 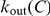 are calculated with crisp memberships, i.e. with 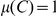 for all members of community 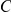.

**Table 4:** Values of 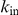 and 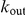 of HLC communities calculated in the bipartite graph of papers and cited sources.

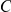	variable	fuzzy 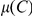	crisp 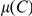
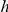 *-index*	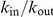	4.94	4.19
	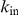	1064.63	1354
	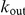	215.37	323
*biblio-*	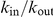	13.03	12.87
*metrics*	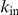	4472.67	4942
	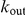	343.33	384
*webo-*	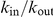	3.46	3.01
*metrics*	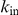	338.29	440
	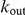	97.71	146

The ratio 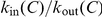, 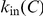, and 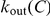 of fuzzy communities found by HLC algorithm calculated with fuzzy and crisp membership grades, respectively, with regard to the bipartite graph.

The assumption that hard clusters can be improved by fitness-based optimisation and fuzzification could not be validated with Ward clusters as input. While the optimised and fuzzified bibliometrics cluster gained slightly better similarity results than the other two algorithms, the clearly identified 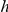-index hard-cluster did not improve because both fitness maximisation and calculating fractional membership grades according to equation 10 included too many nodes which were related but were not assigned to the topic. The fitness-inherent resolution parameter could improve similarity values but would have to be chosen differently for different clusters–a procedure which cannot be applied when target topics are not known in advance. The fact, that fuzzification results in an 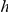-index community with best ratio 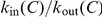 should be interpreted with care. It only means, that the algorithm finds a big cluster which is relatively separated from the rest of the graph. It (partly) includes many papers which do not refer to the 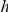-index.

Hierarchical clustering of citation links gave better results than MONC. Link clustering classifies 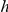-index as a bibliometric topic whereas MONC only includes some 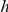-index papers into bibliometrics. Fuzzy cosines of HLC communities and manually selected topics are always better than the corresponding MONC values (s. table 1).

## Discussion

### Assumptions Used

All three algorithms implemented by us are based on the assumptions that a graph's communities (1) are best determined locally, (2) overlap each other, (3) are best described by fractional membership grades, and (4) form a hierarchy. We used these four assumptions as criteria in our selection of approaches to community detection. Nonetheless, with respect to all four criteria there are differences between the selected algorithms. Another criterion was that results should not–at least not strongly–depend on arbitrary parameters. Furthermore, each of the algorithms is based on specific assumptions, which we already mentioned in the respective sections of this paper.

**The fuzzification procedure** based on hard clusters whose fitness is improved assumes that the hierarchical cluster algorithm delivers essentially valid but improvable hard clusters. We did not achieve such an improvement when using standard fitness measures. With respect to its input data, this procedure–like MONC but unlike HLC–assumes that a network of scholarly papers weighted with paper similarity (based on references and/or text) can be used to identify hierarchical thematic structures.

**Hierarchical link clustering:** Paper networks are projections of bipartite graphs and thus do not use the full information content of the raw data. Hierarchical link clustering (HLC) rests on a broader information basis when applied to links in bipartite networks of papers and their cited sources or in tripartite networks of papers, cited sources, and terms used in papers and sources. HLC only assumes that a source is cited for only one reason or for only very few similar reasons in one paper. In the case of terms, the assumption is that authors use one term in one paper with only one meaning. These assumptions are plausible and could be tested in case studies. A further advantage of link clustering is that it enables the combination of citation and textual information in tripartite graphs–a very ‘natural’ solution of this longstanding problem (cf. e.g. the introduction of reference [35] and sources cited there).

For **MONC** there are no further assumptions beyond the four mentioned above and the one about paper-similarity networks. However, we found that MONC needs some post-processing to reveal the hierarchy of a graph. Thus, it is assumed that hierarchical clustering of the nodes' perspectives results in a realistic hierarchy of topics.

### Methodological Aspects

Our implementations of the three approaches to overlapping communities all involve a hard clustering procedure. The fuzzification algorithm uses hard clusters of nodes as input, i.e. clustering has to be done as pre-processing. Hard clustering of fuzzy natural communities is part of MONC's post-processing. In the case of HLC, the algorithm itself is a hard clustering procedure. For HLC we only need to calculate link similarities as some kind of pre-processing.

We have presented results obtained with only one standard hard-cluster algorithm per approach but tested also other ones. Fuzzification and link clustering also worked with Louvain algorithm [36] but we abandoned this fast modularity-driven method due to its use of global information (and the poor hierarchical structure obtained). Fuzzification could perform better with average-linkage clustering but its dendrogram showed only very small stable communities. In the case of average link clustering (HLC) we succeeded in finding relatively stable communities of some size after re-parametrising the dendrogram's similarity axis.

When it comes to defining grades of a node's memberships in different communities, link clustering implies a very plausible and consistent definition. MONC membership grades could also be defined alternatively to the ansatz used here (equation 6). We see this methodological ambiguity as a disadvantage (arbitrary parameters are only a special case of such an ambiguity). In our fuzzification experiments we calculated fractional membership grades using non-fractional (zero or full) membership of nodes as input (equation 10). An alternative would be to use the fitness balances of a node as input for a membership definition. Our attempts to define grades this way led to communities with only very few full members, which could be a desired feature for topic extraction that cannot be achieved by HLC membership grades.

MONC membership grades fit into the framework of fuzzy set theory because a node's grades in general do not sum up to unity. Link clustering leads to node grades which are normalised. Thus, an HLC grade is more adequately interpreted as a probability.

### Concluding Remarks

We implemented three local approaches to the identification of overlapping and hierarchically ordered communities in networks as algorithms and tested their ability to extract manually defined thematic substructures from a network of information-science papers and their cited sources.

Hierarchical clustering of citation links proved to be the most satisfactory approach–with regard to the test results, to its methodological simplicity, to its ability to work with the broadest information basis (the bipartite graph of papers and sources), and to its potential for a simple inclusion of text information in addition to citation data–an issue on top of our agenda.

Clustering citation links does not need to be restricted to a small period of time but can also be applied to a longer time period. This might make it possible to solve the problem of tracing the development of topics over time. The only limitation HLC encounters is the limited coverage of publication databases, i.e. the existence of citation links to publications that are not included in the database.

MONC was found to be useful for overcoming the longstanding problem of field delineation by greedily expanding the paper set downloaded from a citation database [26] (p. 19). Instead of delineating research fields by journal sets, they can be identified with a large enough natural community–obtained with low enough resolution–of an appropriate seed node. In other words, the strictly local approach enables the local exploration of networks which are too big for global analysis like the Web or the complete citation network of scientific papers. A node's natural community is a local structure that can be constructed without knowing the whole graph.

Hierarchical clustering of citation links can be applied to this problem too. Starting with one citation link, we include from its neighbourhood the most similar citation link and proceed in this manner until we reach a large similarity gap.

The fuzzification algorithm matches target topics only in some cases. Iterating fitness-based optimisation may lead to more consistent clusters by removing loosely connected nodes. First experiments did not confirm this assumption. However, there is a large number of variations of how nodes can be included and excluded. An iteration node by node leads to a version of the LFM algorithm [16] applied to hard clusters instead of single nodes or cliques, an approach already proposed by Baumes *et al.* (2005) [23].

A last remark concerns the hierarchical order of networks. Since until now we have only tested whether the methods considered could find communities which correspond to three topics defined before (at different levels of an assumed hierarchy) we did not yet evaluate the whole hierarchies obtained which is a nontrivial issue [37], [38] especially in the case of overlapping communities [16] (p. 6). As for communities, we think of a hierarchy as a structure that can be materialised with more or less certainty.

## Supporting Information

Supporting Information S1 **Basic definitions of fuzzy-set theory and details of algorithms.**
(PDF)
