# Supplementary material for: Identifying Overlapping and Hierarchical Thematic Structures in Networks of Scholarly Papers: A Comparison of Three Approaches
Source: PLoS One. 2012 Mar 27;7(3):e33255. doi: 10.1371/journal.pone.0033255 (PMC3314014; doi:10.1371/journal.pone.0033255)
Supplement: Supporting Information S1 — Basic definitions of fuzzy-set theory and details of algorithms. (PDF) [file pone.0033255.s001.pdf]

Supporting Information for: Identifying Overlapping and Hierarchical Thematic Structures in Networks of Scholarly Papers: A Comparison of Three Approaches

Frank Havemann[1,*] Jochen Gläser[2] Michael Heinz[1] Alexander Struck[1]

November 24, 2011

1 Institut für Bibliotheks- und Informationswissenschaft, Humboldt-Universität zu Berlin, Berlin, Germany
2 Zentrum Technik und Gesellschaft, Technische Universität Berlin, Berlin, Germany
∗ E-mail: Frank (dot) Havemann (at) ibi.hu-berlin.de

Contents

1 Fuzzy Sets 1
1.1 The Characteristic Function 1
1.2 Generalisation of Operations on Sets 2
1.3 Fuzzy Jaccard Index 2
1.4 Fuzzy Cosine Similarity 2
1.5 Fuzzy Precision and Recall 2

2 MONC Algorithm 2
2.1 Pseudo Code 3
2.2 Fitness Function 3
2.3 Resolution Levels 3
2.4 Optimisation 3
2.5 Cliques as Seeds 4

3 Fuzzification Algorithm 4
3.1 Approach 4
3.2 Pseudo Code 4
3.3 Expansion Details 4

1 Fuzzy Sets

The concept of *fuzzy sets* is used to describe gradations of belonging. It was developed by Zadeh (1968) and is a generalisation of the classical set notion. In the case of classical sets we have only to decide whether an element belongs to a set or not. An element of a fuzzy set has a membership grade in the closed interval $[0, 1]$. We generalise set operations like intersection or union for fuzzy sets.

1.1 The Characteristic Function

We start with the definition of the *characteristic function* of a classical set. Let Ω be the *universe* of all possible elements and $\mathbf{M}$ be a subset of Ω.

Definition 1 (Characteristic function) *The function* $\mathbf{M^{Ch}}$ *from* Ω *to the two-value set* $\{0, 1\}$,

$$\mathbf{M^{Ch}} : \Omega \to \{0, 1\},$$

where

$$\mathbf{M^{Ch}}(x) = \begin{cases} 1 & \text{for } x \in \mathbf{M} \\ 0 & \text{otherwise} \end{cases},$$

is called the characteristic function of $\mathbf{M}$.

A classical set $\mathbf{M}$ is uniquely defined by its characteristic function and vice versa. That means, we can identify sets with their corresponding characteristic functions.

To define fuzzy sets we generalise the characteristic function to a function $\mathbf{M}(x)$, called *membership function*, from Ω to the closed interval $[0, 1]$:

$$\mathbf{M} : \Omega \to [0, 1].$$

The membership function $\mathbf{M}(x)$ gives us the *grade of membership* of x in $\mathbf{M}$.

In the paper we denote node's i grade of membership in community C as $\mu_i(C)$.

1.2 Generalisation of Operations on Sets

Definition 2 (Complement) *The complement of a fuzzy set* $\mathbf{M}$ *is the fuzzy set* $\overline{\mathbf{M}}$ *with the membership function*

$$\overline{\mathbf{M}}(x) \doteq 1 - \mathbf{M}(x),$$

where $\mathbf{M}(x)$ *is the membership function of* $\mathbf{M}$.

In the case of classical sets this is a true statement, not a definition, if *membership function* is replaced by *characteristic function*.

We get the membership grade of a node i in the fuzzy complement of a community C by subtracting its grade from one: $1 - \mu_i(C)$.

Intersection and union are generalised in the following definitions.

Definition 3 (Fuzzy Intersection) *The intersection* $\mathbf{M} \cap \mathbf{N}$ *of fuzzy sets* $\mathbf{M}$ *and* $\mathbf{N}$ *is a fuzzy set with the membership function*

$$\mathbf{M} \cap \mathbf{N}(x) = \min\{\mathbf{M}(x), \mathbf{N}(x)\},$$

where $\mathbf{M}(x)$ *and* $\mathbf{N}(x)$ *are the membership functions of* $\mathbf{M}$ *and* $\mathbf{N}$.

We calculate the node's i grade of membership in the intersection of two communities C_1 and C_2 as $\min(\mu_i(C_1), \mu_i(C_2))$.

Definition 4 (Fuzzy Union) *The union* $\mathbf{M} \cup \mathbf{N}$ *of fuzzy sets* $\mathbf{M}$ *and* $\mathbf{N}$ *is a fuzzy set with the membership function*

$$\mathbf{M} \cup \mathbf{N}(x) = \max\{\mathbf{M}(x), \mathbf{N}(x)\},$$

where $\mathbf{M}(x)$ *and* $\mathbf{N}(x)$ *are the membership functions of* $\mathbf{M}$ *and* $\mathbf{N}$.

We calculate the node's i grade of membership in the union of two communities C_1 and C_2 as $\max(\mu_i(C_1), \mu_i(C_2))$.

If elements of Ω can be counted we define a fuzzy set's size as follows:

Definition 5 (Size of a Fuzzy Set) *The size of a fuzzy set* $\mathbf{M}$ *is*

$$|\mathbf{M}| = \sum_{x \in \Omega} \mathbf{M}(x).$$

The size of a fuzzy community C in a graph with n nodes is then

$$|C| = \sum_{i=1}^{n} \mu_i(C).$$

1.3 Fuzzy Jaccard Index

It is defined as the fuzzy analogue of the ordinary Jaccard index. If $\mathbf{M}$ and $\mathbf{N}$ are two fuzzy sets we have to apply the definitions given above to define it as

$$\mathrm{sim}_J(\mathbf{M}, \mathbf{N}) \doteq \frac{|\mathbf{M} \cap \mathbf{N}|}{|\mathbf{M} \cup \mathbf{N}|}.$$

1.4 Fuzzy Cosine Similarity

The same is true for the fuzzy variant of Salton's index also called cosine similarity:

$$\mathrm{sim}_S(\mathbf{M}, \mathbf{N}) \doteq \frac{|\mathbf{M} \cap \mathbf{N}|}{\sqrt{|\mathbf{M}||\mathbf{N}|}}.$$

1.5 Fuzzy Precision and Recall

If $\mathbf{T}$ is the target set and $\mathbf{S}$ the result of search in a database then for both crisp and fuzzy sets precision is defined as

$$\mathrm{precision}(\mathbf{T}, \mathbf{S}) \doteq \frac{|\mathbf{T} \cap \mathbf{S}|}{|\mathbf{S}|}$$

and recall as

$$\mathrm{recall}(\mathbf{T}, \mathbf{S}) \doteq \frac{|\mathbf{T} \cap \mathbf{S}|}{|\mathbf{T}|}.$$

2 MONC Algorithm

The description of MONC given below follows the text in our arXiv paper (Havemann et al. 2010) which was then published in a journal (Havemann et al. 2011). MONC's code in **R** is available from `http://141.20.126.172/~div/code.html`.

We assume that each node is its own natural community G at infinite resolution. The next vertex V from the neighbourhood of G included to G is the one that increases the fitness of G at the largest value of resolution denoted by $\alpha_{\mathrm{incl}}(G, V)$.

2.1 Pseudo Code

In pseudo code the growth of a natural community G can be described as follows ($N(G)$ denotes the neighbourhood of G):

```
while N(G) is not empty do
    for each node V in N(G) do
        calculate α_incl(G, V)
    end for
    include the node with maximum α_incl into G
end while
```

2.2 Fitness Function

If we use the fitness function as defined by Lancichinetti, Fortunato, and Kertesz (2009) a node cannot remain a single because for any α the module fitness of a single is always zero and the module fitness of two neighbours is always larger then zero. We therefore add a one in the numerator of the fitness function:

$$f(G,\alpha) = \frac{k_{\text{in}}(G)+1}{(k_{\text{in}}(G)+k_{\text{out}}(G))^{\alpha}}. \tag{1}$$

This is a minor change. Its influence dissapears for larger communities.

2.3 Resolution Levels

From this definition we can derive a formula for calculating the maximum value of resolution $\alpha_{\text{incl}}(G,V)$, where a node V does not diminish the fitness of a module G when included in it by demanding that for $\alpha < \alpha_{\text{incl}}(G,V)$ we have $f(G\cup V,\alpha) > f(G,\alpha)$:

$$\alpha_{\text{incl}}(G,V) = \frac{\log(k_{\text{in}}(G\cup V)+1) - \log(k_{\text{in}}(G)+1)}{\log k_{\text{tot}}(G\cup V) - \log k_{\text{tot}}(G)}, \tag{2}$$

where $k_{\text{tot}} = k_{\text{in}} + k_{\text{out}}$ denotes the sum of the degrees of all nodes of a module.

We now derive formula 2 for calculating the maximum value of α, where a node V does not diminish the fitness of a module G when included in it. For V in neighbourhood of G we demand therefore

$$f(G\cup V,\alpha) > f(G,\alpha). \tag{3}$$

With definitions given above we then have

$$\frac{k_{\text{in}}(G\cup V)+1}{k_{\text{tot}}(G\cup V)^{\alpha}} > \frac{k_{\text{in}}(G)+1}{k_{\text{tot}}(G)^{\alpha}} \tag{4}$$

and therefore

$$\frac{k_{\text{in}}(G\cup V)+1}{k_{\text{in}}(G)+1} > \left[\frac{k_{\text{tot}}(G\cup V)}{k_{\text{tot}}(G)}\right]^{\alpha}. \tag{5}$$

We take logarithm on both sides of this equation and get

$$\log\frac{k_{\text{in}}(G\cup V)+1}{k_{\text{in}}(G)+1} > \alpha\log\frac{k_{\text{tot}}(G\cup V)}{k_{\text{tot}}(G)}. \tag{6}$$

That means, if $\alpha < \alpha_{\text{incl}}$ with

$$\alpha_{\text{incl}} = \frac{\log(k_{\text{in}}(G\cup V)+1) - \log(k_{\text{in}}(G)+1)}{\log k_{\text{tot}}(G\cup V) - \log k_{\text{tot}}(G)} \tag{7}$$

we have $f(G\cup V,\alpha) > f(G,\alpha)$.

2.4 Optimisation

We can calculate $k_{\text{in}}(G\cup V)$ from $k_{\text{in}}(G)$ and $k_{\text{tot}}(G\cup V)$ from $k_{\text{tot}}(G)$ i.e. the current values of the module from the preceding ones (which saves computing time). For this we define the interaction of a module and a node as

$$k_{\text{inter}}(G,V) = \sum_{i\in G} A_{Vi}, \tag{8}$$

where A denotes the adjacency matrix of the undirected (and in general) weighted graph and calculate the degree of a node or its weight as the sum of the weights of its edges

$$A_{V+} = \sum_i A_{Vi}. \tag{9}$$

The weight of edges of internal nodes k_{in} is increased by $2\cdot k_{\text{inter}}$ because both directions have to be taken into account:

$$k_{\text{in}}(G\cup V) = k_{\text{in}}(G) + 2\cdot k_{\text{inter}}(G,V). \tag{10}$$

The total of all weights is increased by the weights of the edges of the new node:

$$k_{\text{tot}}(G\cup V) = k_{\text{tot}}(G) + A_{V+}. \tag{11}$$

We first include the neighbour V of each node that improves the community's fitness at highest resolution. Then we continue with the new neighbourhood of $G\cup V$ until all nodes are included in the natural community. After each step we compare the current communities of all nodes to find duplicates. Thus we can reduce the number of communities treated by the inclusion algorithm and save further computing time. We merge overlapping natural communities of nodes.

2.5 Cliques as Seeds

If a graph is characterised by a strong variation of its local density and the seed node is located in a high density region, the MONC algorithm immediately leaves this region because it searches for nodes with low degree first. These outside nodes only moderately increase the number of links leaving the community and thus often provide the earliest increase in fitness. We solved this problem by starting from cliques (i.e. totally linked subgraphs) instead of single nodes. Lee *et al.*, who applied the LFK algorithm without the exclusion mechanism, also found that cliques as seeds gave better results than single nodes (Lee, Reid, McDaid, and Hurley 2010).

While Lee et al. (2010) use maximal cliques (i.e. cliques which are not subgraphs of other cliques), we optimise clique size by excluding nodes that are only weakly integrated. Thus, for our starting points we apply an analogue of the LFK exclusion mechanism. In detail, we exclude the node V that diminishes the module fitness at lowest resolution, i.e. has the weakest coupling to the rest of the module G. Analogously to α_{incl} we calculate α_{excl} with

$$\alpha_{\text{excl}}(G, V) = \frac{\log(k_{\text{in}}(G) + 1) - \log(k_{\text{in}}(G \setminus V) + 1)}{\log k_{\text{tot}}(G) - \log k_{\text{tot}}(G \setminus V)}. \tag{12}$$

This procedure is repeated until only two nodes remain in each clique. From the set of shrinking cliques we select the one which is most resistant to further reduction i.e. those with highest α_{excl} of the next node to be excluded. After its exlusion the rest of the clique would be less strongly coupled. That means, we choose the most cohesive subgraph of a clique as optimal.

After optimising all cliques larger than pairs we determine the optimal clique belonging to a seed node by searching for the clique where the seed is member and has its maximum α_{excl}. Nodes which are not member of any optimal clique remain single seeds. Every other node is assigned to one clique, some of them to the same one.

3 Fuzzification Algorithm

3.1 Approach

Our algorithm takes any set of crisp clusters as input. The algorithm evaluates for every node at the border of a cluster (a node with at least one link crossing the crisp border) how the cluster would gain or lose fitness if this node was to be included or excluded. A fitness balance is calculated and if positive the node will be included in the resulting cluster. The experiments have been conducted by computing this balance for every node being in or connected to the hard cluster without actually changing the cluster in question. We add all nodes with a positive fitness balance and remove all with a negative one.

3.2 Pseudo Code

The borders of crisp clusters (i.e. ordinary sets of nodes) in graph G are evaluated:

```
cluster.list ← hierarchical.clustering(A)
for cluster ∈ cluster.list do
    outsiders ← neighbourhood(cluster)
    eval.nodes ← cluster + outsiders
    new.cluster ← ∅
    for V ∈ eval.nodes do
        fit.incl ← Fitness(incl(cluster, V))
        fit.excl ← Fitness(excl(cluster, V))
        fit.balance ← fit.incl − fit.excl
        if fit.balance > 0 then
            new.cluster ← new.cluster + V
        end if
    end for
end for
```

Since the arbitrary parameter α has been set to 1 it doesn't influence the fitness calculation and has therefore been left out here.

3.3 Expansion Details

The three graphs in figure 1 provide an idea of how much each of the selected clusters expanded during fuzzification. The number of nodes in the original hard cluster is shown in red. Nodes that belong to the hard cluster after fitness maximisation are shown in violet. Their membership grade is assumed as being 1 because a decision has been made by the algorithm whether or not to include

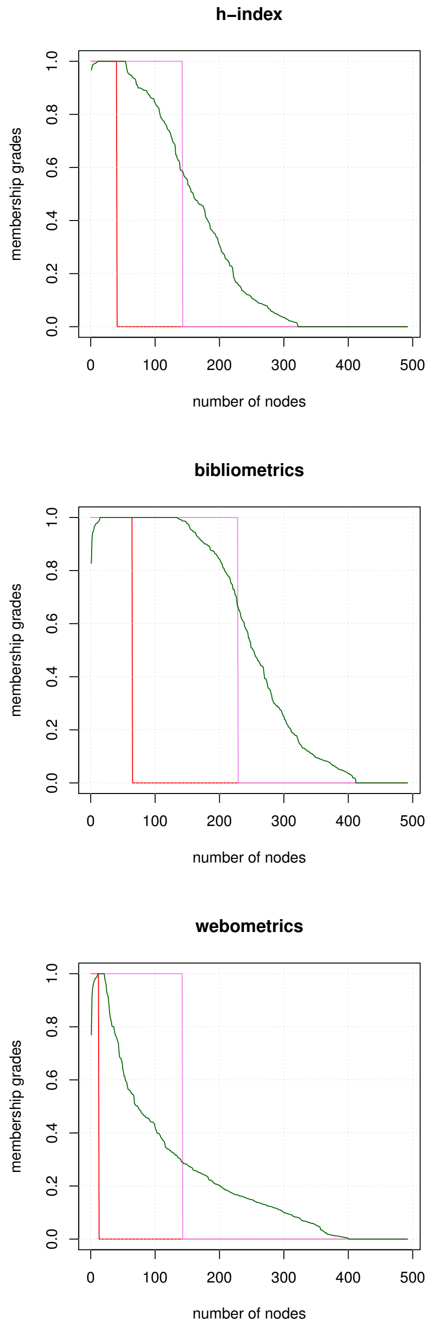

Figure 1: Fuzzy communities related to the three topics (cf. text)

a node. The green curve represents a fuzzy view on the expanded cluster, where the outside border nodes have been taken into account. The membership grade of every node inside the overlapping cluster and directly connected outside nodes are represented in green. The nodes from the original hard cluster have been ordered to the left.

Acknowledgements

This work is part of a project in which we develop methods for measuring the diversity of research. The project is funded by the German Ministry for Education and Research (BMBF). We would like to thank all developers of **R**.[1]

References

Havemann, F., M. Heinz, A. Struck, and J. Gläser (2010). Identification of Overlapping Communities and their Hierarchy by Locally Calculating Community-Changing Resolution Levels. *Arxiv preprint arXiv:1008.1004*.

Havemann, F., M. Heinz, A. Struck, and J. Gläser (2011). Identification of Overlapping Communities and their Hierarchy by Locally Calculating Community-Changing Resolution Levels. *Journal of Statistical Mechanics: Theory and Experiment 2011*, P01023. doi: 10.1088/1742-5468/2011/01/P01023, Arxiv preprint arXiv:1008.1004.

Lancichinetti, A., S. Fortunato, and J. Kertesz (2009). Detecting the overlapping and hierarchical community structure in complex networks. *New Journal of Physics 11*, 033015. arXiv:physics.soc-ph/0802.1218.

Lee, C., F. Reid, A. McDaid, and N. Hurley (2010). Detecting highly overlapping community structure by greedy clique expansion. In *Proceedings of the 4th SNA-KDD Workshop*. ArXiv preprint arxiv:1002.1827.

Zadeh, L. A. (1968). Fuzzy algorithms. *Information and Control 12*, 94–102.

[1] http://www.r-project.org
